# Sleep Quality in Patients with Rheumatoid Arthritis and Associations with Pain, Disability, Disease Duration, and Activity

**DOI:** 10.3390/jcm7100336

**Published:** 2018-10-09

**Authors:** Igor Grabovac, Sandra Haider, Carolin Berner, Thomas Lamprecht, Karl-Heinrich Fenzl, Ludwig Erlacher, Michael Quittan, Thomas E. Dorner

**Affiliations:** 1 Department of Social and Preventive Medicine, Centre for Public Health, Medical University of Vienna, Kinderspitalgasse 15, 1090 Vienna, Austria; igor.grabovac@meduniwien.ac.at (I.G.); thomas.dorner@meduniwien.ac.at (T.E.D.); 2 Department of Rheumatology and Osteology, Kaiser Franz Josef Hospital, Sozialmedizinisches Zentrum Süd, Kundratstrasse 3, 1100 Vienna, Austria; carolin.berner@gmx.net (C.B.); ludwig.erlacher@wienkav.at (L.E.); 3 Karl Landsteiner Institute for Autoimmune Diseases and Rheumatology, Kundratstrasse 3, 1100 Vienna, Austria; t.lamprecht@sportunion.at (T.L.); karlhfenzl@gmail.com (K.-H.F.); 4 Karl Landsteiner Institute for Remobilisation and Functional Health, Kundratstrasse 3, 1100 Vienna, Austria; michael.quittan@wienkav.at; 5 Department of Physical Medicine and Rehabilitation, Kaiser-Franz-Josef-Hospital, Sozialmedizinisches Zentrum Süd, Kundratstrasse 3, 1100 Vienna, Austria

**Keywords:** rheumatoid arthritis, sleep, sleep disorders, pain

## Abstract

We aimed to assess the subjective sleep quality in patients with rheumatoid arthritis (RA) and its correlation with disease activity, pain, inflammatory parameters, and functional disability. In a cross-sectional study, patients with confirmed RA diagnosis responded to a questionnaire (consisting of socio-demographic data, the Health Assessment Questionnaire Disability Index, and the Medical Outcome Study Sleep Scale). Disease activity was assessed with the Clinical Disease Activity Index, and pain levels using the visual analogue scale. In addition, inflammatory markers (C-reactive protein, interleukin-6, and tumor necrosis factor alpha) were analyzed. Ninety-five patients were analyzed, predominantly female, with an average age of 50.59 (9.61) years. Fifty-seven percent reported non-optimal sleep duration, where functional disability (92.7% vs. 69.8%; *p* = 0.006) and higher median pain levels (3.75 (2.3–6.0) vs. 2.5 (2.0–3.5); *p* = 0.003) were also more prevalent. No differences in sociodemographic variables, disease duration or activity, inflammatory parameters, or use of biological and corticosteroid therapy were observed. The multivariate regression analysis showed that more intense pain was associated with a lower likelihood of optimal sleep (odds ratio (OR) = 0.68, 95% confidence interval (CI) 0.47–0.98, *p* = 0.038). Patients with RA report a high prevalence of non-optimal sleep, which is linked to pain level. Clinicians need to be aware of this issue and the potential effects on health and functional status.

## 1. Introduction

Rheumatoid arthritis (RA) is a chronic autoimmune disease characterized by changes in the synovium followed by joint swelling, pain, cartilage and bone destruction, and subsequent systemic inflammation [1]. The exact etiology is still unknown; however, 50% of the risk is attributable to genetics [2]. The incidence of RA has proven difficult to determine due to the wide variety of symptoms with which patients seek medical help and the associated delay in seeking medical help. However, the prevalence of RA has been reported at around 1% globally, with some countries showing reduced prevalence [3]. Overall, the disease is more prevalent in women and has been found to increase in prevalence with age, with the highest rates found in women older than 65 years [2]. Patients with RA have a higher mortality and morbidity burden, reduced quality of life, and higher disability [4].

As in other pain conditions, issues of sleep disturbance are of major concern in RA patients, who often report problems with poor sleep quality, issues with falling asleep, as well as feeling unrested and fatigued after sleep [5,6,7,8]. These subjective findings have been supported by polysomnographic studies where, compared to healthy controls, patients with RA showed lower overall sleep efficiency and more awakenings [9]. Sleep problems in people with chronic illness are associated with a variety of problems, including lower quality of life, psychological issues, cognitive decline, as well as higher morbidity and mortality [10]. In addition, patients with RA have reported that issues with sleep are of high personal importance [6].

A commonly reported symptom of RA is fatigue, which is described as an overwhelming feeling and different from normal tiredness, and is a multifactorial phenomenon associated with sleep issues, pain, depression, and functional limitations [11,12,13]. RA patients that experience fatigue, pain, and depression also have higher levels of physical disability. Several studies have indicated that pain and depression, as well as sleep quality through its relationship with pain and fatigue, are associated with functional disability [7].

Studies have shown that most sleep issues experienced in patients with RA stem from pain. In particular, difficulties with falling asleep and feeling tired after sleep have been found to be associated with lower pain threshold, pain severity, depression, and inflammation in patients with RA [14,15,16,17]. Pain has also been found to predict sleep disturbance over time, even without sleep issues affecting pain. Both pain and sleep issues were found to be associated with depression after a 2-year follow-up [18]. Interestingly, poor clinical management and increased disease activity of RA were found to be associated with lower daytime sleepiness (but also, as expected, with sleep problems), which may be explained by pain-related alertness [19]. However, the exact mechanisms of disease activity and sleep issues are not known. Sleep problems may also be of concern for clinicians working with RA patients, as reduced sleepiness was found in studies of patients taking certain biopharmaceutical therapies [17,20]. For instance, studies on abatacept have shown a positive influence on some aspects of sleep quality [21].

Sleep quality and its associations with disease activity parameters has not yet been researched in Austria. This paper presents the results of a larger cross-sectional study in patients with from RA. The complete study protocol was previously published [22]. The aim of the present study was to determine the prevalence of problems with sleep and its association with disease activity, pain levels, inflammatory parameters, and functional disability in patients with RA.

## 2. Materials and Methods

### 2.1. Participants

One hundred participants visiting the rheumatology outpatient clinic of the Kaiser Franz Josef Hospital in Vienna during their regularly scheduled visits were recruited for the purposes of this study [22]. Inclusion criteria were: age between 18 and 65 years; and fulfilled the RA diagnostic criteria according to the 2010 European League Against Rheumatism (EULAR) classification at the time of inclusion [23]. Additionally, patients with severe comorbidities (fibromyalgia, cancer, severe cardiovascular illness) as well as those that could affect handgrip strength measurements, and patients who refused or were not able to sign the informed consent were excluded from the study. The study took place from November 2015 to August 2016.

### 2.2. Methods

In this monocentric cross-sectional study, patients were approached during their regularly scheduled visits to a rheumatology outpatient clinic, and if they fitted the inclusion criteria, were asked to participate in the study. After informed consent was given, the participants were asked to fill out a questionnaire, after which measurements were taken.

### 2.3. Questionnaire

The questionnaire was made up of four parts and had 49 items in total—multiple choice and open-end questions—and took around 10 min to finish. The questionnaire was designed for self-reporting, with measurements of disease activity undertaken by a member of the study team. The questionnaire was provided in several languages, including German, English, and Turkish, and one with a combination of both Serbian and Croatian.

#### 2.3.1. Socio-Demographic Data

This part consisted of multiple choice and open-end questions and was made up of 13 items covering socio-demographic characteristics of the sample (age, sex, marital status, education level, and current occupation). Additional questions on disease duration, as well as current therapy and comorbidities, were also asked.

#### 2.3.2. Functional Disability

The Health Assessment Questionnaire Disability Index (HAQ-DI) was used to assess the patients’ self-reported functional disability. This validated instrument consists of 20 questions divided into eight categories of functioning: dressing, rising, eating, walking, hygiene, reach, grip, and usual activities. The overall functional disability index is given as a final score, with values between 0 (no functional disability) and 3 (severe functional disability) [24].

#### 2.3.3. Sleep Quality

Quality of sleep was assessed using the Medical Outcome Study Sleep Scale (MOS-SS). This questionnaire is recommended for use in RA patients and is made up of 12 items regarding the patient’s sleep over the last 4 weeks. MOS-SS is a self-report questionnaire that scores six different sleep dimensions as scales and two additional indices: sleep disturbance (four items), daytime somnolence (three items), snoring (one item), awakening short of breath or with a headache (1 item), sleep adequacy (two items), and quantity of sleep (one item, which is not scored, but is an average number of hours spent asleep over the past 4 weeks). Optimal sleep is an added dichotomized variable derived from the quantity of sleep, where sleep is considered optimal if the reported duration is between 7 and 8 h, otherwise it is non-optimal. All items in the MOS-SS, expect for quantity of sleep, are given a numerical score, and the result is the sum of the individual scores, with the minimum value being 0 and the maximum 100. Higher scores indicate more of the named dimension, i.e., more sleep problems. The two indices—sleep problem indices I and II—are derived from several items, with sleep problem index I being derived from six items and sleep problem index II from nine items of the MOS-SS. As with the other items, higher values indicate more sleep problems [25]. Patients were also asked about their use of sleeping pills or pain medication.

### 2.4. Measurements

#### 2.4.1. Disease Activity

Overall disease activity was measured by the Clinical Disease Activity Index (CDAI), which is a widely used and validated instrument. The CDAI score is derived as a sum of the subscales (Swollen 28-Joint Count, Tender 28-Joint Count, Patient Global Disease Activity, and Evaluator’s Global disease Activity). Scores ≤ 2.8 are considered as remission, >2.8 and ≤10 as low disease activity, >10 and ≤22 as moderate, and >22 as high disease activity [26]. For the purposes of our study, we grouped the participants into two categories: remission and low disease activity in one group, and moderate and high disease activity as the other group.

#### 2.4.2. Pain Intensity

Pain was assessed using the visual analogue scale (VAS), ranging from 0 to 10, whereby a higher number indicates a higher pain intensity [27].

#### 2.4.3. Inflammatory Parameters

The inflammatory parameters of C-reactive protein (CRP; mg/dL), interleukin-6 (IL-6; pg/mL), and tumor necrosis factor alpha (TNF-alpha; ng/mL) were obtained through analysis of the patients’ blood samples at the Department of Laboratory Medicine of Kaiser Franz Josef Hospital, where the research was conducted on the same day.

### 2.5. Statistical Analysis

Kolmogorov-Smirnov tests, Shapiro-Wilk tests, and histogram analysis were employed to determine the data distribution. Descriptive statistics were recorded for each variable, with the quantitative variables shown as mean values and standard deviation, and in the case of a non-normal distribution, as median and 25–75 percentile values. Differences between groups were calculated with T-tests or Mann-Whitney U-tests, depending on the data distribution. Chi-square tests were used for the differences between categorical variables. Spearman’s rank order correlation was used for the possible correlations between the dimensions of sleep quality, as derived from the MOS-SS, and the other scores (age, disease duration, pain, CDAI score and HAQ-DI score). A multivariate logistical regression analysis was performed in order to assess the characteristics that were associated with the MOS-SS-derived dichotomized variable of optimal sleep (reported sleep between 7 and 8 h was deemed “optimal”, while reported sleep under 7 h and more than 8 h was deemed “non-optimal”). Variables which showed significant correlations in the univariate correlation analysis were included in the multivariate logistic regression model. All *p*-values under 0.05 were considered statistically significant. The analysis was performed using SPSS for Windows version 24.0 software (IBM, Armonk, NY, USA).

### 2.6. Ethics Approval

The study complied with Good Clinical Practice standards and the Helsinki Declaration. The study was approved by the Ethical Committee of the City of Vienna (number: EK 15-173-0915).

## 3. Results

A total of 140 patients that fulfilled the inclusion criteria were approached and asked to participate. However, 14 (10%) were not interested to take part, 6 (4.3%) declined due to time constraints, 4 (2.9%) didn’t speak any of the languages the surveys were available in and 22 (15.7%) did not complete the necessary measurements, which left 95 (67.9%) patients available for analysis. Approximately two-thirds of the study population were female, with an age range from 22 to 65 years. The mean duration of the disease in our population was around 9 years, with 80% receiving therapy with disease-modifying anti-rheumatic drugs (DMRAD). According to the HAQ-DI score, 21.4% of the participants had a level of functional disability, and the median pain score was 3.0 (2.0–5.0) out of 10. Differences according to optimal sleep duration were found in pain intensity, level of functional disability, and use of non-steroidal anti-inflammatory drugs (NSAID) and disease-modifying drugs. Additional information about the study participants is provided in Table 1.

Overall, our patients reported non-optimal sleep duration in 56.8% of cases, with a mean duration of sleep over the past 4 weeks of 6.5 (1.3) h. The median score of sleep problem index I was 30.0, with the score of sleep problem index II being slightly higher at 32.2 points. The highest reported median value of the six items was in sleep adequacy, which indicates that our patients, in general, feel well rested and have enough sleep during the night. Furthermore, most of the participants did not take pain medication or sleeping pills. Other results regarding the MOS-SS score and the other sleep quality variables are presented in Table 2. In terms of disease activity, patients with moderate and high disease activity reported fewer hours asleep in comparison to patients with low disease activity or in remission (6.7 (1.2) vs. 6.0 (1.2); *p* = 0.009).

The relationship between the sleep domains of the MOS-SS and age, disease duration in months, pain intensity, disease activity, and functional disability scores was investigated, as seen in Table 3. Pain intensity was the only variable that was correlated with all the MOS-SS domains, with the highest coefficient being a medium positive correlation with sleep problem index II (*r* = 0.406; *p* < 0.001). Overall, the strongest relationship was found between functional disability and sleep problem index II (*r* = 0.516; *p* < 0.001). Disease activity had significant small to medium positive correlations with sleep disturbance, snoring, shortness of breath or snoring, and sleep problem indices I and II, while a significant negative correlation was observed between sleep duration and the sleep adequacy domains of the MOS-SS, as seen in Table 3.

According to the multivariate logistical regression analysis, pain intensity was the only variable that was significantly associated with optimal sleep duration, where more severe pain was associated with a reduced likelihood of optimal sleep, as seen in Table 4.

## 4. Discussion

Our study showed that, in an investigated sample of patients with RA, problems with sleep are common, with 56.8% of the participants reporting non-optimal sleep duration. Interestingly, the MOSS-SS sleep adequacy scale showed high results, meaning that our participants were, overall, satisfied with their sleep quality. Studies have shown that up to 70% of patients with RA suffer from problems with sleep, ranging from difficulty falling asleep to difficulty maintaining sleep or suffering from daytime sleepiness [6,28]. Several studies found that higher disease activity is associated with sleep problems [9,29,30]. A study by Wolfe et al. [8] showed that sleep disturbance could be attributed to RA in up to 42% of cases, linking sleep disturbance to pain, mood, and disease activity. Similarly, a Korean study found decreasing subjective sleep quality as the disease activity was increasing [30]. Conversely, studies have also found that reducing the active inflammatory disease and the arthritic process have a positive effect on sleep quality [31]. However, a connection between disease activity and sleep quality has not been universally reported in studies. For example, Hirsch et al. [5] reported overall disturbed sleep in patients with RA, but found no association with inflammatory disease activity. These results have often been questioned as the study population was very small (only 19 patients), which may be the reason for the lack of association. Our study, although five times larger in terms of the sample size, also found no difference in sleep quality in patients with different disease activity, as shown in Table 1. In terms of the relationship between the CDAI scores and the sleep domains of the MOS-SS, seen in Table 3, only low to moderate correlations were found. The only significant difference in sleep characteristics between patients with different disease activity was found in sleep duration, where patients in the moderate/high disease activity group reported shorter sleep duration.

The mechanism of how disease activity influences sleep quality is not completely clear; however, most studies have suggested the connection with joint stiffness and pain. In a study by Wolfe and Michaud [8], pain was shown to be one of the most common underlying reasons leading to problems with sleep, with a recent study of Austrian patients with chronic pain also reporting notable sleep disturbance [32]. Chronic pain and associated sleep issues are a risk for developing depressive symptoms in patients with RA, which in turn may have an additional influence as depression has been reported as being a predictive factor for poor quality of sleep [15,19]. In our study population, pain levels were generally low; however, there was a significant difference based on reported optimal sleep duration, as seen in Table 1, where lower pain levels were found in those patients who reported optimal sleep duration. The multivariate logistic regression model showed pain intensity to be a predictor for non-optimal sleep in our population, as shown in Table 4. Furthermore, pain was found to be correlated with all the domains of sleep quality in the MOS-SS, having a negative relationship with hours spent sleeping and the sleep adequacy domain. More pain intensity is positively correlated with sleep disturbance, snoring and shortness of breath, somnolence, and both sleep problem indices. Recent evidence shows that lower sleep quality lowers the threshold of pain and increases the pain intensity in patients with RA [9,16,19].

The relationship between RA therapy and medication used for sleep quality is ambivalent. Some studies have reported the use of biological medication as having a positive effect on sleep quality, with longitudinal observations of anti-TNF substances also reporting greater sleep quality improvements in patients on abatacept in comparison to methotrexate. A trial investigating the effectiveness of indomethacin on sleep quality showed indomethacin to be superior to placebo in a questionnaire-based study. However, polysomnographic studies investigating sleep patterns in patients using NSAIDs showed no changes in sleep patterns or sleep quality in patients with RA. In our study, as presented in Table 1, patients who reported optimal sleep duration were found to more often use disease-modifying drugs and NSAIDs, which is probably connected to their analgesic effect. More research, especially blinded longitudinal studies, should be done to investigate the effects of medication on sleep quality of RA patients, as most cross-sectional studies have reported no connection [8,30,33].

Functional disability was found to be associated with sleep quality, as patients with RA often experience difficulties with the activities of daily living, which was found to be associated with fatigue and sleep disturbance. In addition, disability often leads to greater depression, which in turn leads to sleep disturbance. A relationship between pain, depression, sleep, and functional disability has been shown in numerous studies, which may mean that there is a casual relationship, as pain, fatigue, and depression inhibit normal daily productivity, causing sleep disorders and disability, which then contribute to pain, fatigue, depression, and disability [8,9,33]. In our study, patients who were categorized as having functional disability reported significantly non-optimal sleep duration, as seen in Table 1. However, the HAQ-DI score showed no association with optimal sleep in the multivariate logistic regression model and, interestingly, was found to have a positive relationship with sleep duration. Furthermore, functional disability was found to be significantly correlated with somnolence, shortness of breath, sleep adequacy, sleep disturbance, and both sleep problem indices as shown in Table 3.

The relationship between age and sleep quality in patients with RA is unclear, but some studies have reported lower sleep quality in older patients [30,33]. Our study showed only a small positive correlation with age and snoring, but no correlation with other dimensions of the MOS-SS as seen in Table 3. Other studies have often failed to report an association between age and sleep problems in patients with RA [8,19]. This was confirmed by our logistic regression model, where there was no significant association found between optimal sleep duration and age.

Finally, the study limitations need to be addressed. The cross-sectional study design does not allow for causal conclusions on the relationships between variables. Longitudinal studies need to be performed in order to examine the possible casual associations. Secondly, although the MOS-SS is recommended for use in RA, it refers to a time frame of the previous 4 weeks, which may affect the results due to recall bias. Additionally, we haven’t reported on the body mass index (BMI) which is also a potential confounder. Finally, as patients were recruited to the study by their physicians, it is possible that the results are an underestimation due to reporting bias. The relatively small sample size might prevent the generalizability of the study results, as well as the higher proportion of women; however, this is to be expected in a RA patient population. Furthermore, our population consisted of men and women of working age, which may also contribute to the reduced sleep problems.

Given our results and the noted associations of pain and non-optimal sleep in patients with RA it is important to alert the clinical community working with RA patients. Sleep as well as pain assessments should be systematically included in the clinical assessments [34]. However, such monitoring will not be effective in reducing the burden of pain and sleep problems in RA patients, unless appropriate treatment is also not provided. Albeit research indicates the associations of pain and sleep disturbances, pharmacotherapy for these comorbidities receives little attention, and there is yet to be a consensus or evidence based treatment algorithms [35]. Additionally, a recent meta-analysis indicated that non-pharmacological sleep treatments (including physiotherapy, meditation, massage, sleep restriction therapy and sleep scheduling, imagery exercises, and others) were associated with large improvements in sleep quality [36]. In conclusion, there is a high frequency of non-optimal sleep duration in patients with RA of working age; however, these patients are mostly satisfied with their overall sleep quality. Our study further shows the association between higher pain levels and non-optimal sleep. Physicians working with RA patients need to be aware of the sleep issues in this population and include pharmacological and psychological interventions as these may have a positive effect on sleep quality, and in turn on psychological wellbeing, physical wellbeing, and functional disability. Future studies could focus on sleep quality in newly diagnosed patients, as well as longitudinal studies need to be implemented in order to see if improvements in sleep contribute to reduction of depression, pain, and functional disability.

## Figures and Tables

**Table 1:** Sociodemographic and disease-related variables stratified by optimal sleep duration.

Variable	Total (*n* = 95)	Non-Optimal Sleep Duration (*n* = 54)	Optimal Sleep Duration (*n* = 41)	*p*
**Age**; mean (SD)	50.59 (9.61)	49.98 (9.14)	51.39 (10.25)	0.482
**Sex**				
Male	32.6%	33.3%	31.7%	0.867
Female	67.4%	66.7%	68.3%	
**Relationship status**				
In a relationship	73.7%	77.8%	68.3%	0.298
Not in a relationship	26.3%	22.2%	31.7%	
**Education level**				
Primary level	15.8%	13.0%	19.5%	0.434
Secondary level	72.6%	77.8%	65.9%	
Tertiary level	11.6%	9.3%	14.6%	
**Employment status**				
Employed	61.1%	63.0%	58.5%	0.661
Unemployed	38.9%	37.0%	41.5%	
**Disease duration** in months; median (Q_25_–Q_75_)	72.0 (36.0–141.0)	78.0 (36.0–144.0)	60.0 (28.50–138.0)	0.452
Pain intensity; median (Q_25_–Q_75_)	3.0 (2.0–5.0)	3.75 (2.3–6.0)	2.5 (2.0–3.5)	0.003
**Functional disability**				
No disability	79.8%	69.8%	92.7%	0.006
Disability	20.2%	30.2%	7.3%	
**Disease activity**				
Remission	29.5%	29.6%	30.8%	0.726
Low	33.7%	31.5%	38.5%	
Moderate	26.3%	27.8%	25.6%	
High	8.4%	11.1%	5.1%	
**Inflammatory parameters**				
CRP (mg/dL); median (Q_25_–Q_75_)	3.20 (1.10–6.70)	3.05 (1.07–7.05)	2.70 (1.00–5.55)	0.594
TNF-α (g/mL); median (Q_25_–Q_75_)	1.60 (0.56–2.35)	1.65 (0.54–2.89)	1.60 (0.64–2.32)	0.884
IL-6 (pg/mL); median (Q_25_–Q_75_)	3.89 (1.98–7.91)	4.47 (2.04–9.67)	3.44 (1.72–6.18)	0.179
**Therapy**				
Disease-modifying drugs	81.1%	72.2%	92.7%	0.012
Biologicals	43.2%	50.0%	34.1%	0.122
Corticosteroid	16.8%	18.5%	14.6%	0.616
Non-steroidal anti-inflammatory	14.7	7.4%	24.4%	0.021
Other medication	55.8%	55.6%	56.1%	0.958

SD = standard deviation. Differences between groups were calculated with the T-test, Mann-Whitney U test dependent on data distribution. Chi-square test was used for differences between categorical variables.

**Table 2:** Sleep characteristics of the study participants.

Sleep-Related Variables	*n* = 95
Sleep disturbance median (Q_25_–Q_75_)	32.5 (15.0–51.2)
Snoring; median (Q_25_–Q_75_)	40.0 (20.0–60.0)
Shortness of breath or headache; median (Q_25_–Q_75_)	20.0 (0.0–30.0)
Sleep adequacy; median (Q_25_–Q_75_)	60.0 (30.0–80.0)
Somnolence; median (Q_25_–Q_75_)	26.6 (13.3–46.6)
Sleep problem index I; median (Q_25_–Q_75_)	30.0 (16.6–46.6)
Sleep problem index II; median (Q_25_–Q_75_)	32.2 (18.3–47.8)
**Optimal sleep**	
Yes	43.2%
No	56.8%
**Pain medication for sleep**	
Daily	8.2%
Up to 3 times a week	7.2%
More than 3 times a week	5.2%
Up to 3 times a month	12.4%
Never	67.0%
**Sleeping pills**	
Daily	9.3%
Up to 3 times a week	2.1%
More than 3 times a week	1.0%
Up to 3 times a month	4.1%
Never	83.5%
**Hours asleep;** mean (SD)	6.5 (1.3)

SD = standard deviation.

**Table 3:** Spearman’s rank order correlation of variables that correlate with the sleep domains.

Variable	Hours Asleep	Sleep Disturbance	Snoring	Awakening Short of Breath or with Headache	Sleep Adequacy	Sleep Somnolence	Sleep Problem Index I	Sleep Problem Index II
Age	0.077	−0.007	0.204 *	−0.012	0.043	−0.027	−0.050	−0.040
Disease duration	−0.177	0.201 *	−0.037	0.134	−0.066	0.184	0.173	0.149
Pain intensity (VAS)	−0.350 **	0.379 **	0.228 *	0.279 **	−0.297 **	0.305 **	0.374 **	0.406 **
CDAI Score	−0.304 **	0.324 **	0.210 *	0.249 *	−0.275 **	0.148	0.293 **	0.322 **
HAQ-DI Score	0.247 *	0.459 **	0.090	0.298 **	−0.436 **	0.390 **	0.495 **	0.516 **

* p < 0.005; ** *p* < 0.001; VAS: visual analogue scale, CDAI: Clinical Disease Activity Index, HAQ-DI: Health Assessment Questionnaire Disability Index.

**Table 4:** Logistic regression model of variables associated with optimal sleep duration.

Variable	OR	95% CI	*p*
Age	1.03	0.99–1.09	0.162
Pain VAS scale	0.68	0.47–0.98	0.038
HAQ-DI score	0.53	0.19–1.45	0.215
CDAI score	1.02	0.95–1.11	0.544
Disease duration	1.00	0.99–1.01	0.756

OR: odds ratio, 95% CI: 95% confidence interval, VAS: visual analogue scale, CDAI: Clinical Disease Activity Index, HAQ-DI: Health Assessment Questionnaire Disability Index.
